# Prognosis comparison in chronic kidney disease stages 4–5 patients with low parathyroid hormone level due to different causes

**DOI:** 10.1186/s12882-026-05202-7

**Published:** 2026-07-16

**Authors:** Xiao Zhang, Junzhe Chen, Yi Lu, Sha Fu, Chun Ye, Hai Huang, Qiang Liu, Sheng Nie, Ying Tang

**Affiliations:** 1 Department of Nephrology, The Third Affiliated Hospital of Southern Medical University, Guangzhou, China; 2 National Clinical Research Center for Kidney Disease, State Key Laboratory of Organ Failure Research, Guangdong Provincial Institute of Nephrology, Guangdong Provincial Key Laboratory of Renal Failure Research, Division of Nephrology, Nanfang Hospital, Southern Medical University, Guangzhou, China

**Keywords:** Low parathyroid hormone, Prognosis, Chronic kidney disease, Etiology, Cohort study

## Abstract

**Background:**

Existing studies have focused on secondary hyperparathyroidism with elevated PTH in CKD, but survival outcomes of CKD patients with low PTH remain underexplored. This study examines prognostic implications of PTH reduction from different etiologies.

**Methods:**

This multicenter retrospective cohort study included CKD stages 4–5 patients with PTH < 150 pg/mL from the China Renal Data System, categorized into parathyroidectomy (PTX), Drug, and Unknown Causes (UC) groups based on low PTH etiology. Mortality was analyzed using Kaplan-Meier curves and Cox regression. Covariates were selected by univariate or LASSO regression. Inverse probability weighting was applied in sensitivity analyses to reduce confounding.

**Results:**

A total of 17 175 patients were included: 423 in the PTX group, 10 519 in the Drug group, and 6 233 in the UC group. PTX group had the highest survival rate (79.44%) and lowest cardiovascular mortality (1.61%), followed by Drug group (69.21% and 4.60%) and UC group (50.76% and 9.83%) (all *P* < 0.01). Both Drug group and UC group were consistently associated with significantly higher mortality risk compared with the PTX group (both *P* < 0.05). After applying IPW to balance key covariates, PTX group remained associated with the lowest mortality risk (both *P* < 0.05). However, in some sensitivity analyses, no significant difference was observed between PTX group and Drug group.

**Conclusions:**

Pharmacological therapy and PTX were associated with more favorable survival profiles than low PTH of unknown causes in patients with advanced CKD, though residual confounding due to baseline differences between groups cannot be fully excluded.

**Supplementary Information:**

The online version contains supplementary material available at https://doi.org/10.1186/s12882-026-05202-7.

## Background

Chronic kidney disease-mineral and bone disorder (CKD-MBD) is characterized by elevated parathyroid hormone (PTH) and imbalance in calcium (Ca) and phosphorus. The prevalence of severe high PTH level among hemodialysis patients was 15% [1]. 93% of US dialysis patients receive oral phosphorus binders to lower phosphorus levels toward the normal range [2]. CKD-MBD mainly manifested as CKD-associated osteoporosis, which increases fracture risk in CKD patients and CKD-associated cardiovascular disease, including vascular calcification and structural abnormalities [3]. As CKD-MBD affects both the skeletal and cardiovascular systems, reducing the survival rate and quality of life in CKD patients, the prognosis of CKD-MBD has received increasing attention in recent years [4–6].

In the management of CKD-MBD patients, most research has focused on secondary hyperparathyroidism, particularly the impact of elevated PTH level on patients. There is a consensus that high PTH is associated with poorer prognoses in CKD patients [7–9]. In contrast, there has been relatively limited investigation into the clinical outcomes of CKD patients with low PTH level.

Both parathyroidectomy (PTX) and pharmacological interventions can significantly reduce PTH levels in CKD patients. Additionally, certain patients with diabetes, malnutrition, or infections may also exhibit low PTH levels without PTX or medication. However, the prognostic implications of hypoparathyroidism resulting from different underlying causes remain unclear.

To address the above problems, we conducted a multicentric retrospective cohort study recruited patients with CKD stages 4–5 and PTH lower than 150 mg/mL from China Renal Data System database (CRDS). Based on the various etiologies of low PTH levels, the CKD stages 4–5 patients were divided into three groups: PTX group, Drug group and unknown causes (UC) group. This study aims to compare the risks of all-cause mortality, cardiovascular mortality among these three groups.

## Methods

### Data source

This multicentric retrospective cohort study was conducted based on the CRDS, including 556 402 CKD patients from 24 clinical centers, covering the period from January 2000 to March 2022. The CRDS collects routine clinical data from inpatients, covering demographics, vital signs, medications, lab tests, surgical history, and comorbidities. These data, gathered from multiple centers, were harmonized and curated at the National Clinical Research Center for Kidney Disease in Guangzhou, China. Previous research has validated the accuracy and comprehensiveness of this database [10–12].

### Study population

We included CKD inpatients aged ≥ 18 years, with at least one record of PTH in the database. CKD was identified using the International Classification of Diseases, 10th Revision (ICD-10) code N18, or based on an estimated glomerular filtration rate (eGFR) < 60mL/min/1.73 m², urinary albumin-to-creatinine ratio (UACR) ≥ 30 mg/g, sustained for a minimum of 90 days.

Exclusion criteria included: (1) PTH ≥ 150pg/mL; (2) eGFR ≥ 30mL/min/1.73 m² (3) pregnancy at or before the index date; (4) patients diagnosed with malignant tumors; (5) patients diagnosed with severe infection; and (6) patients who were followed for < 30 days.

### Exposure and outcome

Participants were categorized according to their medication prescriptions and surgical history. Patients with low PTH after PTX were divided into the PTX group; Patients with low PTH after medication prescriptions including: cinacalcet, paricalcitol, alfacalcidol, calcitriol, vitamin D, calcium supplement with vitamin D, sevelamer, lanthanum carbonate were divided into the Drug group, which never experienced PTX. Others patients without PTX or using the aforementioned medication were divided into the UC group. In CRDS, patients were enrolled during hospitalization, and subsequent follow-up data were obtained from outpatient visits or readmissions. For patients with documented PTX or medication use, the index date was defined as the date of the first post-intervention PTH < 150 pg/mL. Thus, follow-up began after the intervention, and all PTX or Drug patients had already received treatment at enrollment. For UC group (no documented intervention), the index date was the date of first detected PTH < 150 pg/mL. No patient in UC group underwent PTX or drug therapy during follow-up.

The index date was defined as the earliest recorded PTH level of < 150pg/mL in the database. The primary outcome was all-cause mortality. The secondary outcome included cardiovascular mortality, cardiovascular events, cerebrovascular events, and combined cardiovascular or cerebrovascular events. Cardiovascular events encompassed newly developed unstable angina pectoris, myocardial infarction, and heart failure, while cerebrovascular events included cerebral infarction and cerebral hemorrhage.

### Covariates

Demographic data, medical history, medication usage, and laboratory results were obtained from the CRDS, where the former two categories were recorded prior to the index date. Blood pressure, weight, and height—used to compute body mass index (BMI) as weight divided by height squared—were derived from medical records maintained at each clinical center.

Hypertension was defined by ICD-10 code I10. Diabetes mellitus (DM) was determined by the ICD-10 code E11 or any use of antidiabetic medications. Cardiovascular diseases included previously diagnosed acute coronary syndrome (myocardial infarction or unstable angina) (ACS), stable angina before the index date. Cerebrovascular diseases included previously diagnosed cerebral hemorrhage and cerebral infarction before the index date.

### Statistical analysis

R version 4.1.1 (R Foundation for Statistical Computing, Vienna, Austria) was used to perform all analyses. A two-sided P value < 0.05 was considered the threshold for statistical significance. Multiple imputation was performed using the mice package in R with 5 imputations, 50 iterations, predictive mean matching, and a fixed seed (500). The imputation model included all variables in the final analysis. Cox regression was fitted to each imputed dataset, and results were pooled using Rubin’s rules. Comparisons of normally distributed continuous variables were conducted using analysis of variance, while non-normally distributed continuous variables were assessed with the Kolmogorov–Smirnov test. Data with a symmetric distribution are presented as mean ± standard deviation (SD), whereas those with an asymmetric distribution are reported as median, interquartile range. Kaplan-Meier analysis was used to compare all-cause mortality, cardiovascular mortality and incidence of cardiovascular and cerebrovascular events between different groups.

Survival outcomes were analyzed using the Kaplan–Meier method. A P value of < 0.05 was considered statistically significant. Cox regression was used to assess the association between study groups and all-cause mortality. Covariates with <25% missing data were included in univariate Cox analysis, and those with *P* < 0.05 were entered into the multivariable model. Hazard ratios (HR) with a 95% confidence interval (CI) were estimated after adjusting for age, gender, systolic blood pressure (SBP), diastolic blood pressure (DBP), dialysis status, hypertension, diabetes, ACS, heart failure, arrhythmia, stroke, peripheral vascular disease (PVD), fracture, PTH, Ca, alkaline phosphatase (ALP), N-terminal pro-B-type natriuretic peptide (NT-proBNP), creatinine (Cr), blood urea nitrogen (BUN), Cystatin C, serum albumin, total protein, glucose, low-density lipoprotein cholesterol (LDL), cholesterol, triglyceride, hemoglobin, Drug group and UC group taking PTX group as reference. To address overfitting and multicollinearity, LASSO regression was performed to select key predictors. Variables with non-zero coefficients were included in the final multivariable Cox model to estimate adjusted HR for all-cause mortality.

### Sensitivity analyses

Besides the multivariable Cox regression analysis, we conducted eight sensitivity analyses to assess the robustness of our main findings. (1) Inverse probability weighting (IPW): 8 key covariates (age, sex, hypertension, diabetes, dialysis status, history of ACS, stroke, and heart failure) were included in the propensity score model, and stabilized weights were applied to balance their distribution across groups. Standardized mean differences (SMDs) were calculated to assess balance after weighting; covariates with SMD > 0.1 were additionally adjusted for in the subsequent weighted multivariable Cox proportional hazards models. (2) Patients in PTX group were matched with Drug group and UC group in a ratio of 1:3:3 based on age, since PTX group had the smallest number of subjects and served as the reference for matching. (3) A complete case analysis was performed that excluded all individuals with missing data. (4) the study population was restricted to individuals without history of cardiovascular diseases. (5) The study population was limited to individuals with follow-up time ≥ 180 days. (6) Using alternative thresholds (< 70 pg/mL and < 110 pg/mL) in non-dialysis CKD stages 4–5 patients were performed to test robustness based on KDOQI 2003 guidelines (PTH target: 70–110 pg/mL for stage 4, 150–300 pg/mL for stage 5 or dialysis) [13]. (7) Population was limited to individuals with persistently low PTH (< 150 pg/mL) at 30 days after the initial measurement. (8) Restricting the analysis to patients with persistently low PTH after 30 days whose measurements were obtained during outpatient visits.

This study was reported following the Strengthening the Reporting of Observational Studies in Epidemiology (STROBE) guideline for cohort studies.

## Results

### Patient characteristics

**Fig. 1:**
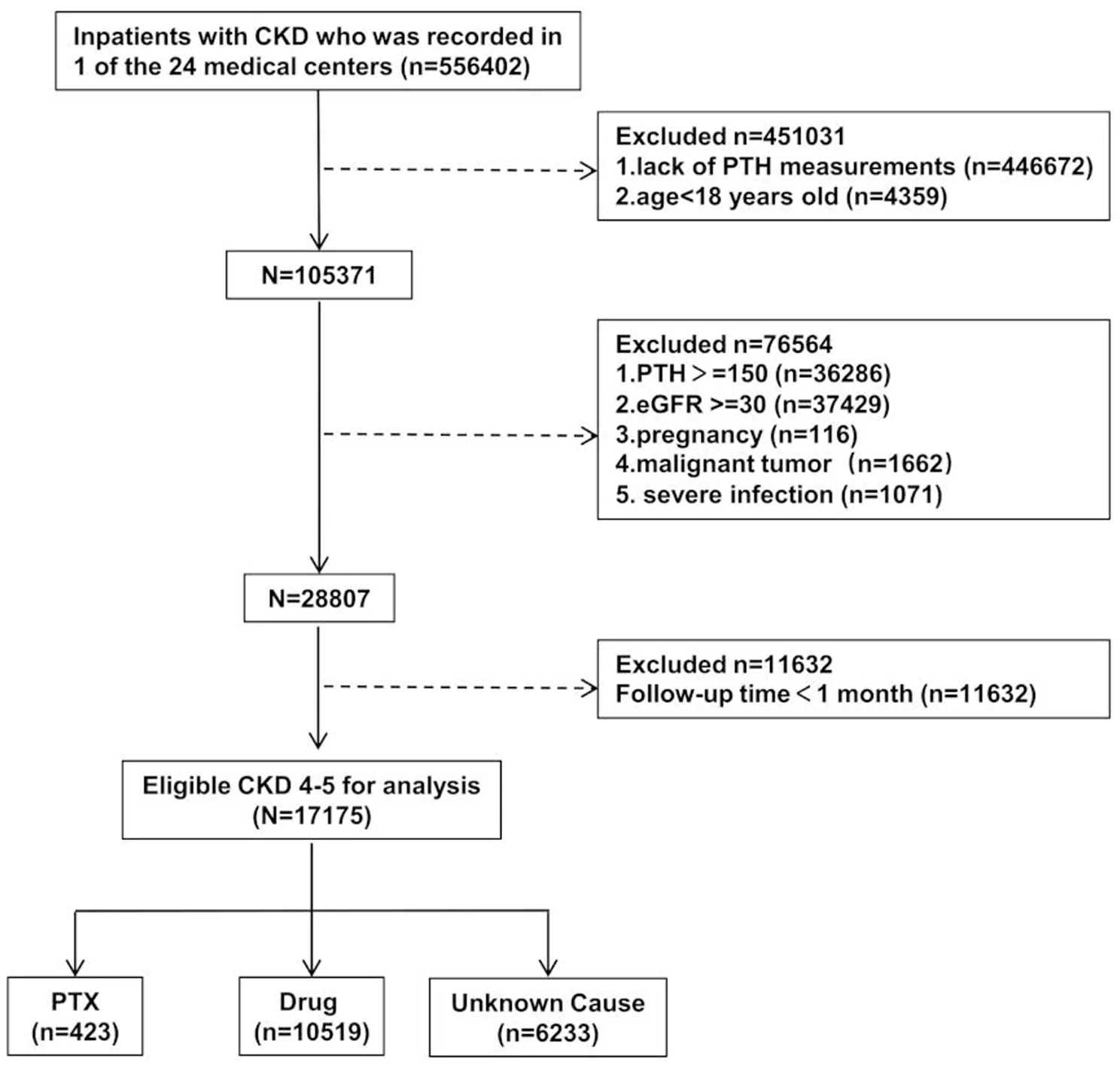
Flow chart of study population. Patients were divided into PTX, Drug, and UC groups based on low PTH etiology. Abbreviations: CKD, chronic kidney disease; PTH, parathyroid hormone; PTX, parathyroidectomy

As shown in Fig. 1, this study included 17 175 patients with CKD stages 4–5. The patients were categorized according to medication prescriptions and surgical history: 423 in PTX group, 10 519 in Drug group, and 6 233 in UC group. Patients in PTX group were younger; had a higher proportion of female, lower SBP, had higher proportion of hypertension, heart failure and fracture, but a lower prevalence of diabetes, ACS, and stroke. Additionally, they had higher baseline levels of phosphorus, ALP, Cr, albumin and hemoglobin, but lower levels of intact PTH, NT-proBNP, uric acid (UA), 24 h proteinuria, glycosylated hemoglobin (HbA1c) (Table 1). UC group displayed generally opposite characteristics, while Drug group showed an intermediate profile. Missing data proportions for each variable are provided in Supplementary Table 1. Compared with PTX and Drug groups, UC group had higher levels of selected inflammatory markers (C-reactive protein (CRP), interleukin-6 (IL-6), and white blood cell count (WBC)) (Supplementary Table 2), a higher prevalence of diabetes, and lower serum albumin. However, UC group did not have the lowest BMI, nor did it have the highest prevalence of hypertension, malnutrition or history of fracture across the three groups.

**Table 1:** Baseline characteristics of participants among CKD stages 4–5 patients with PTH < 150pg/mL

Characteristics	PTX(*n* = 423)	Drug(*n* = 10519)	UC(*n* = 6233)	*P* value	SMD
Demographics					
age, years	50.00 (11.32)	56.99 (15.60)	61.38 (15.10)	< 0.001	0.55
male, %	202 (47.8)	5996 (57.0)	4018 (64.5)	< 0.001	0.227
SBP, median (IQR), mmHg	137.00 [122.00, 152.00]	144.00 [130.00, 160.00]	145.00 [129.00, 162.00]	< 0.001	0.247
DBP, median (IQR), mmHg	84.00 [74.00, 91.00]	83.00 [75.00, 93.00]	81.00 [72.00, 91.00]	< 0.001	0.093
BMI, median (IQR), kg/m^2^	21.83 [19.56, 24.34]	22.44 [20.20, 25.09]	22.57 [20.37, 25.22]	0.017	0.059
Follow-up duration, median (IQR), days	721[344, 1188]	584[232, 1121]	380[132, 884]	< 0.001	0.219
Comorbidities, (%)					
dialysis status	394(93.1)	5690(54.1)	1858(29.8)	< 0.001	1.070
hypertension	268 (63.4)	5660 (53.8)	2636 (42.3)	< 0.001	0.286
diabetes	34 (8.0)	2761 (26.2)	1716 (27.5)	< 0.001	0.351
ACS	33 (7.8)	1194 (11.4)	712 (11.4)	0.071	0.082
heartfailure	79 (18.7)	1841 (17.5)	751 (12.0)	< 0.001	0.123
hyperlipidemia	39 (9.2)	1183 (11.2)	508 (8.2)	< 0.001	0.070
stroke	20 (4.7)	1117 (10.6)	561 (9.0)	0.003	0.149
fracture	30 (7.1)	243 (2.3)	81 (1.3)	< 0.001	0.198
malnutrition	11 (2.6)	198 (1.9)	117 (1.9)	0.563	0.033
Laboratory parameters, median (IQR)					
PTH, pg/mL	22.91 [7.90, 55.89]	85.70 [48.86, 118.20]	84.59 [51.14, 115.10]	< 0.001	0.74
P, mmol/L	1.63 [1.24, 2.12]	1.46 [1.19, 1.80]	1.39 [1.15, 1.68]	< 0.001	0.266
Ca, mmol/L	2.23 [1.89, 2.47]	2.23 [2.08, 2.39]	2.20 [2.07, 2.33]	< 0.001	0.139
ALP, IU/L	205.50 [122.21, 432.75]	72.00 [57.00, 93.00]	76.00 [59.75, 98.00]	< 0.001	0.644
NT-proBNP, pg/mL	191.14 [66.68, 529.36]	279.00 [83.75, 887.45]	243.50 [83.20, 880.60]	0.045	0.095
Cr, µmol/L	875.00[671.30, 1080.80]	572.00 [368.60, 821.00]	428.70 [285.00, 662.00]	< 0.001	0.816
UA, µmol/L	393.80 [321.25, 475.00]	412.00 [332.00, 504.00]	446.00 [354.00, 543.00]	< 0.001	0.288
24 h proteinuria, g/24 h	0.82 [0.34, 2.06]	2.24 [0.95, 4.40]	1.97 [0.74, 4.22]	< 0.001	0.381
albumin, g/L	37.10 [33.30, 40.75]	35.40 [30.70, 39.30]	34.90 [30.30, 38.90]	< 0.001	0.297
Hba1c, %	5.50 [5.00, 5.90]	5.80 [5.20, 6.60]	6.00 [5.40, 7.00]	< 0.001	0.415
cholesterol, mmol/L	4.16 [3.55, 4.98]	4.34 [3.56, 5.29]	4.21 [3.45, 5.16]	< 0.001	0.118
triglyceride, mmol/L	1.54 [1.02, 2.20]	1.47 [1.03, 2.16]	1.47 [1.03, 2.09]	0.158	0.086
hemoglobin, g/L	99.00 [84.00, 115.75]	96.00 [81.00, 113.00]	95.00 [80.00, 112.00]	< 0.001	0.117

Continuous variables were presented as mean (SD) or median (25th percentile to 75th percentile). Categorical variables were expressed as number (percentage). The percentages of comorbidities were be retained one decimal place to better visualize the distribution differences between groups. Abbreviations: PTX, parathyroidectomy; UC, unknown causes; IQR, interquartile range; SMD, standardized mean differences; SBP, systolic blood pressure; DBP, diastolic blood pressure; BMI, body mass index; ACS, acute coronary syndrome; PTH, parathyroid hormone; P, phosphorus; Ca, calcium; ALP, alkaline phosphatase; NT-proBNP, N-terminal pro-B-type natriuretic peptide; Cr, creatinine; UA, uric acid; HbA1c, glycosylated hemoglobin

### Endpoints

**Fig. 2:**
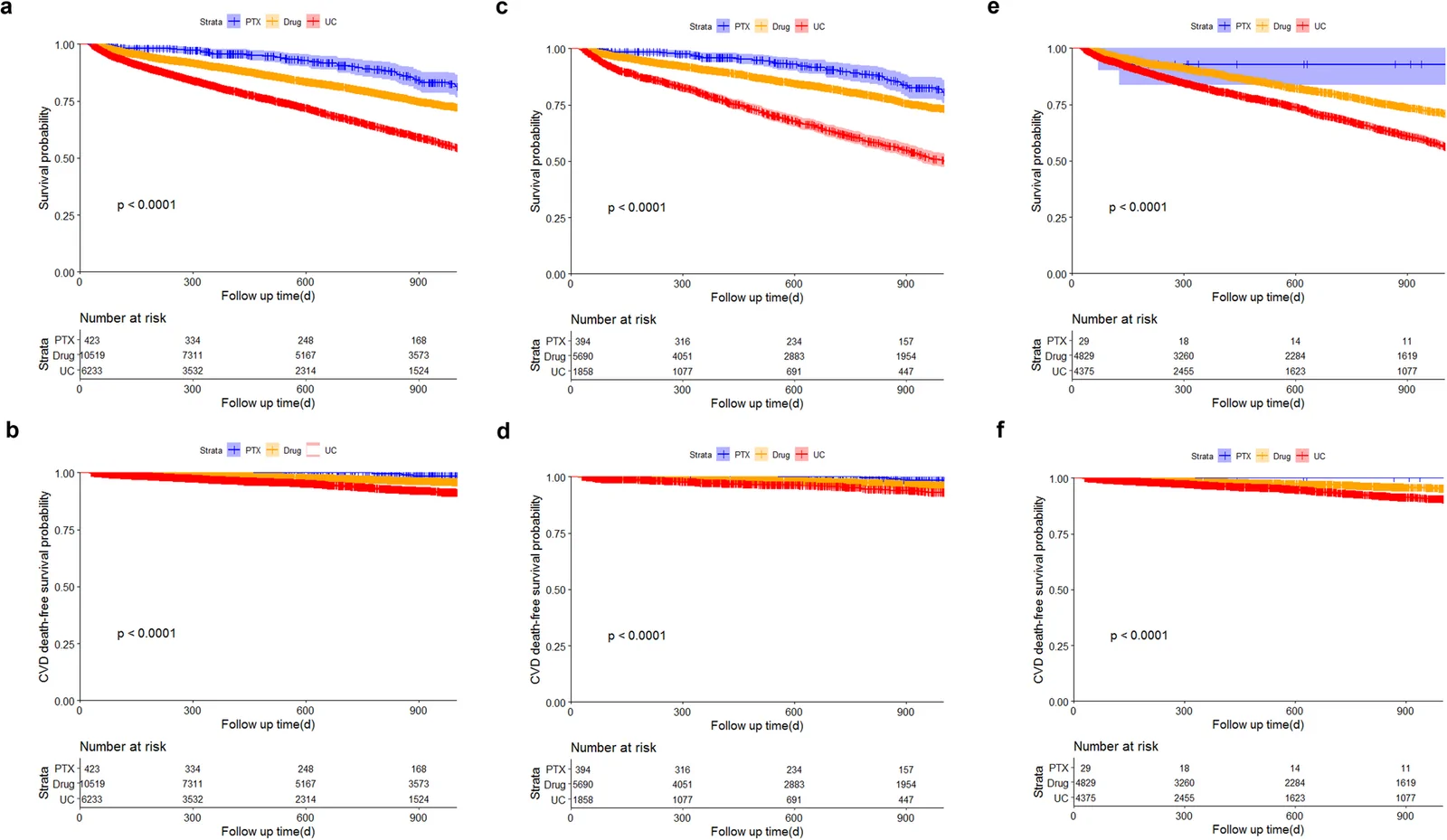
Kaplan-Meier survival curves. Kaplan-Meier curves for all-cause and cardiovascular mortality in overall (**a**, **b**), dialysis (**c**, **d**), and non-dialysis (**e**, **f**) subgroups. Abbreviations: PTX, parathyroidectomy; UC, unknown causes, CVD cardiovascular desease

Kaplan-Meier survival curves were analyzed the survival for patients with CKD stages 4–5, categorized into different groups (Fig. 2). PTX group had significantly higher survival rate than other groups, while UC group had the lowest survival rate (PTX 79.44% vs. Drug 69.21% vs. UC 50.76%, *P* < 0.01). Similarly, regarding cardiovascular death outcomes, PTX group recorded the lowest mortality rate (PTX 1.61% vs. Drug 4.60% vs. UC 9.83%, *P* < 0.01) (Table 2). The absolute numbers of all-cause death, cardiovascular death, cardiovascular events, and cerebrovascular events during 3-year follow-up are presented in Supplementary Table 3. Given the substantial alterations in pathophysiology and serum biochemistry induced by dialysis in patients with CKD stages 4–5, we stratified the cohort into two subgroups based on dialysis status. The results demonstrated that regardless of dialysis, patients who underwent PTX exhibited the lowest all-cause mortality (dialysis, PTX 21.28% vs. Drug 29.70% vs. UC 53.59%, *P* < 0.01; non-dialysis, PTX 7.16% vs. Drug 32.12% vs. UC 47.27%, *P* < 0.01)and cardiovascular mortality (dialysis, PTX 1.71% vs. Drug 3.96% vs. UC 8.02%, *P* < 0.01; non-dialysis, PTX 0% vs. Drug 5.36% vs. UC 10.59%, *P* < 0.01) within this population, followed by Drug group, whereas UC group manifested the highest mortality rate (Table 2; Fig. 2). Additionally, new cardiovascular or cerebrovascular events occurred less frequently in the PTX group than other groups (Supplementary Fig. 1).

**Table 2:** 3-year Mortality rate among CKD stages 4–5 patients with PTH < 150pg/mL

	Group	all-cause mortality rate (%)	95%CI	CVD mortality rate (%)	95%CI
CKD 4–5	PTX	20.56	15.14–25.63	1.61	0-3.40
	Drug	30.79	29.60-31.96	4.60	4.01–5.18
	UC	49.24	47.44–50.97	9.83	8.60-11.05
dialysis subgroup	PTX	21.28	15.78–26.77	1.71	0-3.64
	Drug	29.70	28.11–31.29	3.96	3.22–4.69
	UC	53.59	50.44–56.74	8.02	5.93–10.10
Non-dialysis subgroup	PTX	7.16	0-16.74	0	0–0
	Drug	32.12	30.35–33.88	5.36	4.44–6.29
	UC	47.27	45.14–49.39	10.59	9.09–12.09

Abbreviations: CI, confidence interval; CVD, cardiovascular disease; PTX, parathyroidectomy; UC, unknown causes

### Cox proportional hazards regression

The covariates included in the univariate and multivariable Cox regression analyses are presented in Table 3. Cox proportional hazards regression model demonstrated that age, male, DBP, history of DM, heart failure, stroke, ALP, NT-proBNP, BUN, Cystatin C, total protein, glucose and cholesterol were positively associated with mortality (all *P* < 0.05). Conversely, SBP, PTH, Cr, albumin, LDL and hemoglobin were the protective factors in patients with CKD stages 4–5 and PTH<150 mg/mL (all *P* < 0.05). Moreover, when PTX group served as the reference, the HR in Drug group and UC group was 1.31(95%CI 1.04–1.66, *P* = 0.02) and 2.05 (95%CI 1.61–2.60, *P* < 0.01), respectively.

**Table 3:** Cox regression analysis for factors related to all- cause death

Parameters	Univariate analysis		Multivariate analysis		
	HR (95%CI)	*P*	HR (95%CI)		*P*
age	1.69(1.64–1.74)	<0.001	1.032(1.029–1.034)	<0.001	
male	1.14(1.08–1.2)	<0.001	1.101(1.040–1.164)	<0.001	
SBP	1.06(1.03–1.08)	<0.001	0.998(0.997–0.999)	0.046	
DBP	0.88(0.86–0.91)	<0.001	1.006(1.003–1.009)	0.002	
dialysis status	0.87(0.82–0.91)	<0.001	0.977(0.915–1.043)	0.481	
hypertension	1.14(1.08–1.19)	<0.001	0.980(0.917–1.048)	0.562	
diabetes	1.58(1.5–1.67)	<0.001	1.200(1.120–1.285)	<0.001	
ACS	1.6(1.48–1.72)	<0.001	0.975(0.894–1.064)	0.574	
heartfailure	1.62(1.52–1.73)	<0.001	1.260(1.162–1.363)	<0.001	
arrhythmia	1.59(1.42–1.77)	<0.001	1.051(0.934–1.182)	0.409	
hyperlipidemia	1.09(1-1.19)	0.062			
stroke	1.6(1.48–1.73)	<0.001	1.172(1.078–1.275)	<0.001	
PVD	1.52(1.27–1.83)	<0.001	0.908(0.745–1.106)	0.337	
malnutrition	1.08(0.88–1.32)	0.448			
obesity	0.92(0.78–1.08)	0.319			
fracture	1.47(1.26–1.73)	<0.001	1.087(0.919–1.285)	0.330	
PTH	0.96(0.94–0.98)	0.002	0.9991(0.9984–0.9998)	0.012	
P	1.01(0.99–1.04)	0.397			
Ca	0.84(0.82–0.87)	<0.001	0.916(0.813–1.033)	0.152	
ALP	1.05(1.03–1.07)	<0.001	1.0007(1.0004–1.0009)	<0.001	
CK	0.97(0.93–1.01)	0.127			
NT-proBNP	1.14(1.12–1.17)	<0.001	1.000015(1.000012–1.000018)	<0.001	
Cr	0.84(0.82–0.86)	<0.001	0.9997(0.9996–0.9998)	<0.001	
BUN	1.03(1-1.05)	0.029	1.008(1.004–1.011)	<0.001	
Cystatin C	0.97(0.95-1)	0.038	1.036(1.024–1.048)	<0.001	
UA	0.99(0.97–1.02)	0.554			
albumin	0.74(0.72–0.76)	<0.001	0.952(0.947–0.958)	<0.001	
total protein	0.88(0.86–0.9)	<0.001	1.012(1.009–1.016)	<0.001	
glucose	1.09(1.07–1.11)	<0.001	1.009(1.002–1.015)	0.010	
LDL	0.96(0.93–0.99)	0.004	0.952(0.908–0.998)	0.041	
cholesterol	0.96(0.93–0.99)	0.004	1.042(1.004–1.081)	0.031	
triglyceride	0.93(0.91–0.96)	<0.001	0.990(0.967–1.014)	0.419	
hemoglobin	0.8(0.78–0.82)	<0.001	0.994(0.993–0.996)	<0.001	
cause of low PTH					
PTX(Reference)	Reference		Reference		
Drug	1.34(1.1–1.65)	0.004	1.310(1.036–1.657)	0.024	
UC	2.41(1.97–2.96)	<0.001	2.048(1.614-2.600)	<0.001	

Abbreviations: HR, hazard ratio; CI, confidence interval; SBP, systolic blood pressure; DBP, diastolic blood pressure; ACS, acute coronary syndrome; PVD, peripheral vascular disease; PTH, parathyroid hormone; P, phosphorus; Ca, calcium; ALP, alkaline phosphatase; CK, creatione phosphate kinase; NT-proBNP, N-terminal pro-B-type natriuretic peptide; Cr, creatinine; BUN, blood urea nitrogen; UA, uric acid; LDL, low-density lipoprotein cholesterol; PTX, parathyroidectomy; UC, unknown causes

**Fig. 3:**
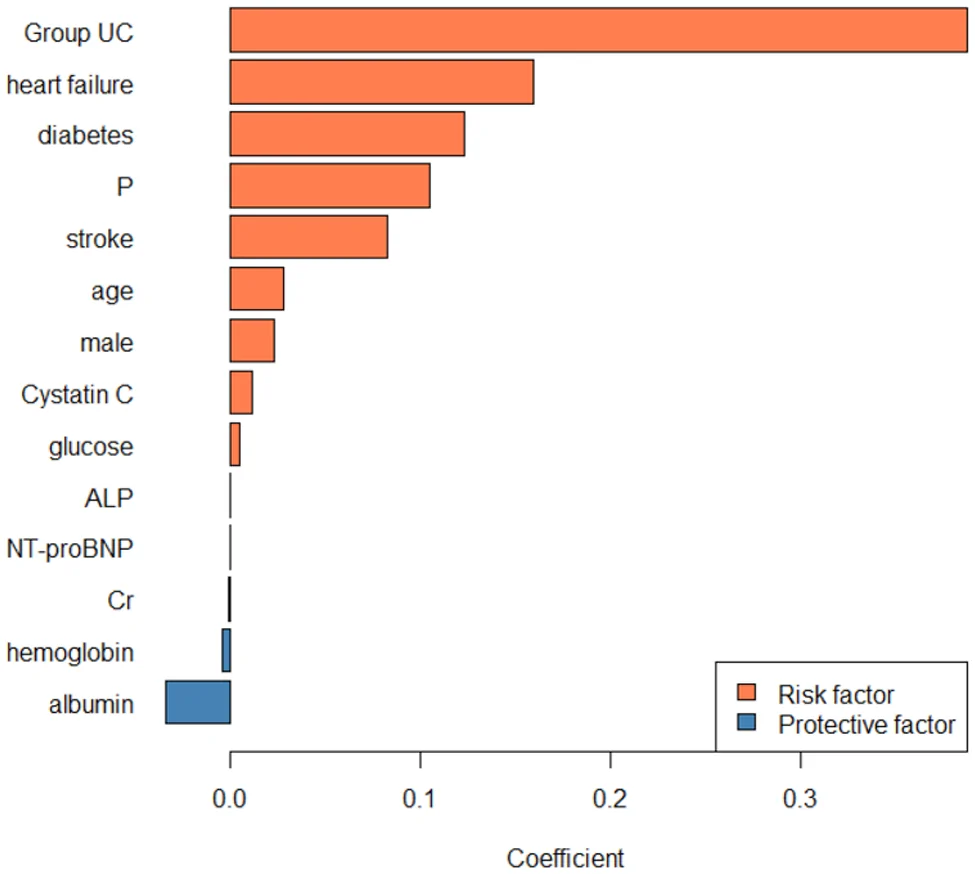
Coefficient plot of variables with non-zero coefficients at the optimal λ in LASSO regression analysis. Abbreviations: UC, unknown causes; P, phosphorus; ALP, alkaline phosphatase; NT-proBNP, N-terminal pro-B-type natriuretic peptide; Cr, creatinine

LASSO regression with 10-fold cross-validation identified variables with non-zero coefficients at the optimal λ (Supplementary Fig. 2). UC group, history of DM, heart failure, stroke, age, male, NT-proBNP, phosphorus, cystatin C, ALP and glucose were risk factors, while Cr, albumin, and hemoglobin were protective factors (Fig. 3). These variables were entered into the multivariable Cox model. Compared with PTX group, Drug group (HR 1.29, 95% CI 1.03–1.62, *P* = 0.03) and UC group (HR 2.02, 95% CI 1.61–2.54, *P* < 0.01) showed significantly higher mortality risk (Table 4).

**Table 4:** Multivariable Cox regression analysis based on LASSO-selected variables

Variable	Coefficient	HR (95%CI)	*P*
age	0.0284	1.032(1.029–1.034)	<0.001
male	0.0238	1.122(1.064–1.183)	<0.001
diabetes	0.1238	1.141(1.074–1.212)	<0.001
heartfailure	0.1596	1.220(1.138–1.307)	<0.001
stroke	0.0829	1.178(1.086–1.278)	<0.001
P	0.1054	1.305(1.234–1.379)	<0.001
ALP	0.0004	1.0007(1.0005–1.0010)	<0.001
NT-proBNP	0.00002	1.000020(1.000017–1.000022)	<0.001
Cr	− 0.0001	0.9995(0.9994–0.9996)	<0.001
Cystatin C	0.0118	1.035(1.027–1.045)	<0.001
albumin	− 0.0338	0.962(0.958–0.967)	<0.001
glucose	0.0049	1.011(1.005–1.017)	<0.001
hemoglobin	− 0.0037	0.995(0.993–0.996)	<0.001
cause of low PTH			
PTX(Reference)	Reference	Reference	
Drug	0	1.293(1.031–1.622)	0.026
UC	0.3880	2.020(1.608–2.538)	<0.001

Variables with non-zero coefficients identified by LASSO regression (10-fold cross-validation) were entered into the multivariable Cox proportional hazards model. The optimal penalty parameter λ was selected based on minimum partial likelihood deviance. Abbreviations: P, phosphorus; ALP, alkaline phosphatase; NT-proBNP, N-terminal pro-B-type natriuretic peptide; Cr, creatinine; PTX, parathyroidectomy; UC, unknown causes

The proportional hazards assumption was tested using Schoenfeld residuals for the final model. The global test was significant (*P* < 0.01), indicating a potential violation. Therefore, a 300-day landmark analysis was performed, in which the PH assumption was fully satisfied (group *P* = 0.188; global *P* = 0.212). Results remained consistent (Supplementary Table 4).

**Fig. 4:**
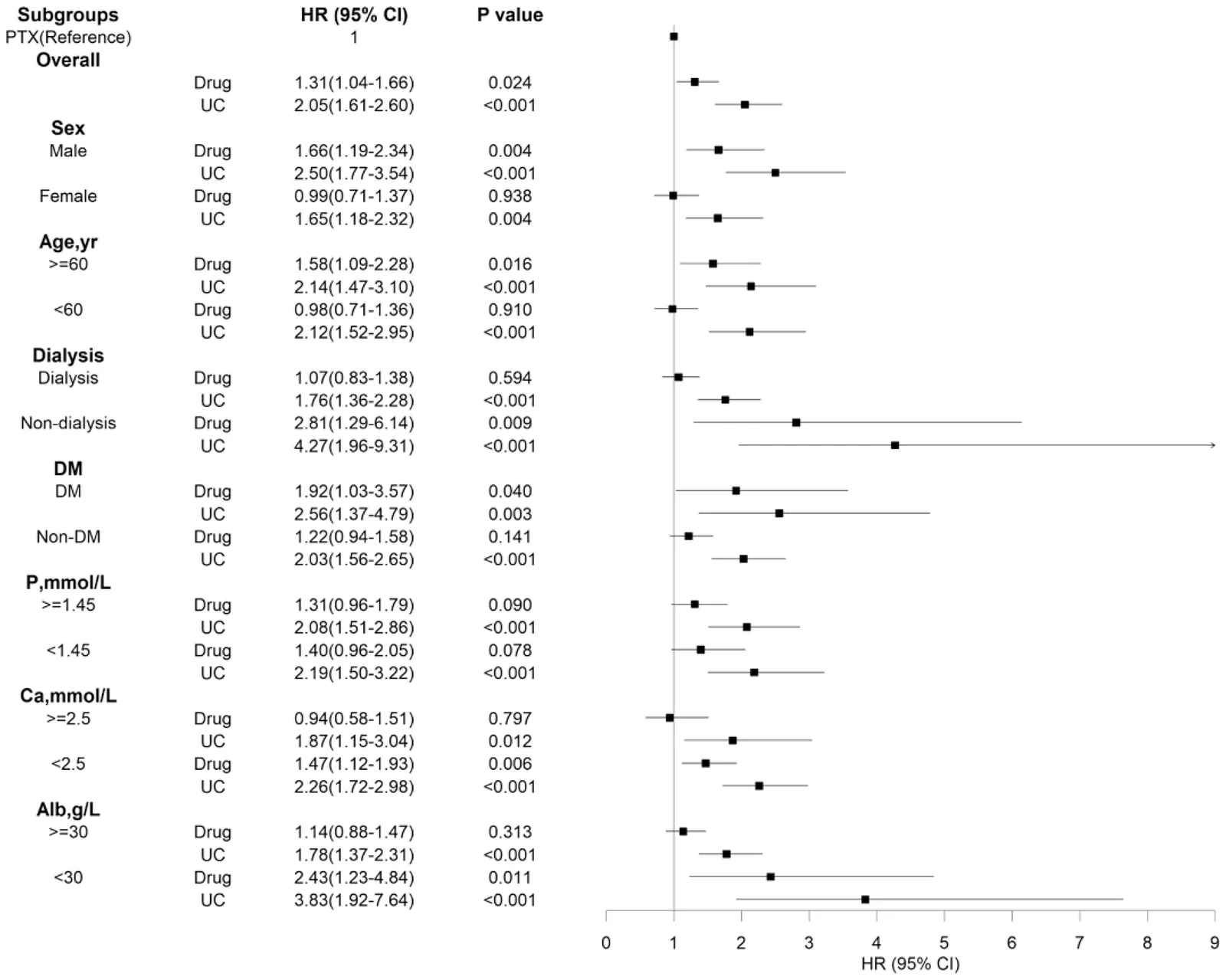
Subgroup analysis. Hazard ratios (95% CIs) for PTX vs. Drug and PTX vs. UC. Abbreviations: PTX, parathyroidectomy; UC, unknown causes; DM, diabetes mellitus; P, phosphorus; Ca, calcium; Alb, albumin

We also conducted subgroup analyses, which revealed that the PTX group had a lower risk of mortality compared with Drug group in male patients over 60 years old, without dialysis or hypercalcemia, with history of DM or hypoproteinemia (all *P* < 0.05, shown in Fig. 4). Interestingly, the PTX group consistently showed a significantly lower mortality risk than the UC group across all subgroups (all *P* < 0.05). To explore heterogeneity within Drug group, we performed exploratory subgroup analyses based on medication type (calcimimetics, vitamin D and its analogs, and phosphate binders). The results showed that calcimimetics users showed favorable survival compared to PTX and significantly better outcomes than UC; vitamin D and its analog users had worse outcomes compared to PTX but remained better than UC; phosphate binder users showed no significant difference from PTX and were better than UC (Supplementary Fig. 3).

### Sensitivity analyses

The results of sensitivity analyses are presented in Table 5. (1) Due to substantial differences in age, sex, and comorbidities across groups, we performed a sensitivity analysis using IPW to balance key covariates: age, sex, hypertension, diabetes, dialysis status, history of ACS, stroke, and heart failure. The balance of covariates after IPW is presented in Supplementary Table 5. Most covariates achieved adequate balance (SMD < 0.1), with the exception of hypertension, diabetes and stroke (SMD 0.23, 0.21 and 0.16, respectively), which were further adjusted in the weighted multivariable Cox models. The results showed that PTX group remained associated with the lowest risk of mortality after balancing (both *P* < 0.05). (2) The Cox proportional hazards regression model, after 1:3:3 age matching showed that the risk of death in UC group remained significantly higher than PTX group (*P* < 0.01). (3) When excluding patients with a history of cardiovascular diseases, the HR of all-cause death in PTX group was significantly lower compared to UC group (*P* < 0.01). (4) In the complete case analysis, or (5) when patients with fewer than 180 days of follow-up were excluded, the risk of death remained significantly higher in UC group compared to the PTX group (both *P* < 0.01). In contrast, no statistically significant difference was observed between the PTX group and the Drug group in the latter four sensitivity analyses. (6) Sensitivity analyses using alternative thresholds (< 70 pg/mL and < 110 pg/mL) in non-dialysis CKD populations yielded consistent results: UC group had the poorest prognosis, while PTX and pharmacological therapy were associated with better outcomes. (7) In analyses restricted to patients with persistently low PTH after 30 days, UC group remained at a higher mortality risk compared with PTX group (HR 1.57, 95% CI 1.04–2.36, *P* = 0.03), whereas no significant difference was observed between Drug and PTX groups (HR 1.29, 95% CI 0.87–1.92, *P* = 0.21) (Supplementary Fig. 4 and Supplementary Table 6). Furthermore, no significant survival difference was observed between patients with persistently low PTH and those with non-low PTH (≥ 150 pg/mL) across the three groups. (8) When further restricted to those with persistently low PTH after 30 days whose measurements were obtained during outpatient visits, the findings were similar (UC vs. PTX: HR 1.60, 95% CI 1.03–2.46, *P* = 0.04; Drug vs. PTX: HR 1.35, 95% CI 0.88–2.06, *P* = 0.17) (Supplementary Fig. 5 and Supplementary Table 7).

**Table 5:** Sensitivity analysis of the association between Drug group, UC group and mortality using PTX group as the reference

model		HR (95%CI)	*P* value
	PTX(Reference)	1	-
overall(base model)	Drug	1.310(1.036–1.657)	0.024
	UC	2.048(1.614-2.600)	<0.001
inverse probability weighting	Drug	2.536(1.174–3.062)	0.011
	UC	4.156(1.971–5.116)	<0.001
age matcahing with 1:3:3	Drug	1.014(0.745–1.381)	0.927
	UC	2.212(1.619–3.023)	<0.001
complete case analysis	Drug	1.356(0.971–1.893)	0.073
	UC	2.174(1.549–3.051)	<0.001
excluding patients with CVD	Drug	1.140(0.871–1.492)	0.340
	UC	1.903(1.451–2.496)	<0.001
follow-up ≥ 180 days	Drug	1.130(0.860–1.484)	0.377
	UC	1.923(1.460–2.534)	<0.001
PTH < 110 pg/mL in non-dialysis	Drug	3.083(1.388–6.955)	0.007
	UC	4.510(2.007–10.137)	<0.001
PTH < 70 pg/mL in non-dialysis	Drug	3.216(1.376–7.515)	0.007
	UC	4.638(1.985–10.836)	<0.001
30days PTH persistently low	Drug	1.289(0.866–1.918)	0.212
	UC	1.567(1.041–2.359)	0.031
outpatients with persistently low PTH	Drug	1.349(0.882–2.064)	0.168
	UC	1.595(1.032–2.464)	0.036

Inverse probability weighting (IPW): weighted Cox proportional hazards model was adjusted for inverse probability weighting weights. Abbreviations: HR, hazard ratio; CI, confidence interval; PTX, parathyroidectomy; UC, unknown causes; CVD, cardiovascular disease

## Discussion

In this observational cohort of patients with CKD stages 4–5 and PTH < 150 pg/mL, low PTH arising from different etiologies was associated with distinct prognoses. Patients in UC group had the poorest outcomes, whereas patients in Drug and PTX groups had more favorable survival profiles. Low PTH arising from various reasons have distinct prognoses which should be recognized and treated differently in clinical setting.

Adynamic or low-turnover bone with a marked decrease in active remodeling sites appears when the level of PTH is too low [14]. Previous studies have shown that patients with low PTH showed worse clinical outcomes, including higher risks of all-cause death and cardiovascular death in dialysis patients [15–17]. This progressive decline in survival may be attributed to PTH’s multifaceted role in bone metabolism and cardiovascular health [18]. Low bone turnover impairs calcium-buffering capacity, which has been linked to accelerated cardiovascular calcification [19]. In our cohort, we lack bone-specific data (bone turnover markers, bone biopsy, or vascular calcification measurements). Thus, while low PTH has been linked to adynamic bone disease and cardiovascular mortality in previous studies, our mechanistic interpretations remain speculative and should be viewed with caution.

However, emerging evidence showed that the higher risk of mortality associated with very low PTH was most evident among patients only when patients had not received any treatment for secondary hyperparathyroidism [20, 21]. There was a nominally significant reduction in the primary cardiovascular endpoint including mortality in the cinacalcet treatment group in whom serum PTH was better controlled than in the placebo treatment group [22, 23]. Low PTH levels, even when falling below current guideline targets, do not necessarily increase fracture risk and may instead be associated with a reduced risk of fracture [24]. An analysis was demonstrated that only among patients who had not undergone PTX were observed the association between increased mortality and low PTH [25]. On the other hand, PTH levels alone may not necessarily reflect patient prognosis. Calcium and phosphorus levels should also be taken into account [26]. Furthermore, a study has shown that total ALP is a better predictor of mortality and fracture risk in hemodialysis patients than PTH [27].

PTX and pharmacological interventions effectively reduce PTH in CKD patients as treatments for secondary hyperparathyroidism. Pharmacological interventions encompass calcimimetics [7, 28, 29], vitamin D [30] and its analogs, and so on. PTX remains a treatment option [31], with the advantage of obviating drug titration and improving bone mineral density [32]. Some studies suggest that PTX may be associated with favorable outcomes [33–35]. However, large-scale randomized controlled trials are still needed to confirm the survival benefit of PTX compared with pharmacological therapy. Drug group was heterogeneous. Broadly, calcimimetic was associated with favorable outcomes, whereas vitamin D and its analogs were associated with poorer prognosis. Notably, the 2009 KDIGO guideline set a PTH target of 2 to 9 times the upper normal limit (approximately 130–585 pg/mL for PTH), which was primarily based on studies using vitamin D analogs. In this specific population with relatively low PTH, calcimimetic or PTX may not increase mortality risk. However, this hypothesis requires confirmation in future large-scale prospective studies.

Some advanced CKD patients, including those on dialysis, exhibit persistently low PTH without PTX or pharmacological interventions. These patients are biologically heterogeneous: low PTH in the UC group may reflect malnutrition, inflammation, diabetes, frailty, adynamic bone disease, or other unmeasured mechanisms rather than a single disease entity [20, 36–40]. In our study, UC group had higher inflammatory markers (CRP, IL-6, WBC), lower albumin, and a higher prevalence of diabetes, supporting the possibility that systemic illness and metabolic heterogeneity partly contributed to their poorer outcomes. However, we lacked data on bone turnover, frailty, nutritional assessment, and vascular calcification. Therefore, UC category should be interpreted as a clinically useful but mechanistically mixed group, and future studies should further distinguish the underlying causes of low PTH in this population.

To ensure the robustness of our findings, we conducted eight sensitivity analyses. UC group consistently had significantly higher mortality risk than PTX group. Although PTX group showed lower mortality risk than Drug group, the difference was not statistically significant in some sensitivity analyses, suggesting that the relative prognosis of PTX and pharmacological therapy should be interpreted cautiously. In conclusion, patients with low PTH of unknown causes had the poorest prognosis, whereas pharmacological therapy and PTX were associated with more favorable outcomes. However, residual confounding cannot be fully excluded.

The limitations of this study are as follows: (1) Despite the use of IPW and multivariable adjustment, confounding by indication and residual confounding cannot be fully excluded. Patients in PTX group were younger and clinically different from those in Drug and UC groups, and some important covariates remained imbalanced even after weighting. Therefore, the observed survival advantage associated with PTX should be interpreted as an association rather than evidence of a causal benefit, and our findings are hypothesis-generating, requiring prospective validation. (2) PTX group had a significantly smaller sample size, as most patients prefer pharmacological therapy due to concerns over surgical complications and the need for a multidisciplinary team, which prolongs hospitalization and increases costs. To reduce this discrepancy, we performed 1:3:3 age-matching and IPW, which supported the robustness of our main findings. Nevertheless, residual selection bias cannot be completely ruled out, and larger multi-center prospective studies are warranted. (3) we defined low PTH as < 150 pg/mL based on current guidelines. However, the optimal PTH target remains unclear, particularly for non-dialysis CKD stages 4–5 patients, representing an important direction for future research. (4) the underlying causes of low PTH in UC group were unclear and potentially multifactorial due to missing data, so categorizing them as a single group may be overly simplistic. (5) patients with both medication use and PTX were classified into the PTX group, as they likely had suboptimal responses to pharmacological therapy, with PTX being the primary reason for PTH reduction. (6) We lack data on drug doses, treatment duration, adherence, and time-varying treatment changes. These unmeasured factors may introduce bias, and our findings should be interpreted with caution. (7) Several key variables, including NT-proBNP, HbA1c, and proteinuria, had substantial missing data. Although multiple imputation was performed, the high missingness may have introduced bias.

## Conclusions

The parathyroid glands primarily regulate homeostasis of calcium and phosphate through the secretion of PTH. Even with aggressive pharmacological management of PTH, calcium and phosphorus levels, other factors related to hyperparathyroidism may continue to contribute to systemic damage in CKD patients. The complex direct and indirect actions of CKD-MBD make it imperative for future studies to uncover more specific biomarkers indicative of disease progression or treatment effectiveness. In this observational study, pharmacological therapy, particularly calcimimetic use, and PTX were associated with more favorable survival profiles than low PTH of unknown causes. However, the findings should be interpreted cautiously because residual confounding due to baseline differences between treatment groups cannot be fully excluded.

## Supplementary Information

Below is the link to the electronic supplementary material.

Supplementary Material 1

## Data Availability

The datasets used and analysed during the current study are available from the corresponding authors on reasonable request.

## Abbreviations

ACS: Acute coronary syndrome
ALP: Alkaline phosphatase
BMI: Body mass index
BUN: Blood urea nitrogen
Ca: Calcium
CI: Confidence interval
CKD: Chronic Kidney Disease
CKD-MBD: Chronic kidney disease-mineral and bone disorder
Cr: Creatinine
CRDS: China Renal Data System database
CRP: C-reactive protein
DBP: Diastolic blood pressure
DM: Diabetes mellitus
eGFR: Estimated glomerular filtration rate
HbA1c: Glycosylated hemoglobin
HR: Hazard ratios
IL-6: Interleukin-6
IPW: Inverse probability weighting
LDL: Low-density lipoprotein cholesterol
NT-proBNP: N-terminal pro-B-type natriuretic peptide
P: Phosphorus
PVD: Peripheral vascular disease
PTH: Parathyroid Hormone
PTX: Parathyroidectomy
SBP: Systolic blood pressure
SD: Standard deviation
SMDs: Standardized mean differences
UA: Uric acid
UC: Unknown Causes
WBC: White blood cell count
