# Supplementary material for: Prognosis comparison in chronic kidney disease stages 4–5 patients with low parathyroid hormone level due to different causes
Source: BMC Nephrol. 2026 Jul 16;27:606. doi: 10.1186/s12882-026-05202-7 (PMC13660033; doi:10.1186/s12882-026-05202-7)
Supplement: Supplementary file 1 [file 12882_2026_5202_MOESM1_ESM.doc]

**Supplementary Tables**

**Supplementary Table 1. Missing data proportions for each variable.**

| Variable | Missing, n (%) |
| --- | --- |
| age | 0 (0%) |
| sex | 0 (0%) |
| SBP | 2803 (16.3%) |
| DBP | 2762 (16.1%) |
| BMI | 0 (0%) |
| Follow-up duration | 0 (0%) |
| dialysis status | 0 (0%) |
| hypertension | 0 (0%) |
| diabetes | 0 (0%) |
| ACS | 0 (0%) |
| heartfailure | 0 (0%) |
| hyperlipidemia | 0 (0%) |
| stroke | 0 (0%) |
| fracture | 0 (0%) |
| malnutrition | 0 (0%) |
| PTH | 0 (0%) |
| P | 1122 (6.5%) |
| Ca | 6 (0) |
| ALP | 976 (5.6%) |
| NT-proBNP | 6888 (40.1%) |
| Cr | 0 (0%) |
| UA | 35 (0.2%) |
| 24h proteinuria | 7920 (46.1%) |
| albumin | 28 (0.1%) |
| Hba1c | 7271 (42.3%) |
| cholesterol | 393 (2.3%) |
| triglyceride | 1242 (7.2%) |
| hemoglobin | 227 (1.3%) |

Abbreviations: SBP, systolic blood pressure; DBP, diastolic blood pressure; BMI, body mass index; ACS, acute coronary syndrome; PTH, parathyroid hormone; P, phosphorus; Ca, calcium; ALP, alkaline phosphatase; NT-proBNP, N-terminal pro-B-type natriuretic peptide; Cr, creatinine; UA, uric acid; HbA1c, glycosylated hemoglobin.

**Supplementary Table 2.** Inflammatory and infection-related laboratory parameters.

| Laboratory parameters of inflammation and infection, median (IQR) | PTX  (n=423) | Drug  (n=10519) | UC  (n=6233) | P value | SMD |
| --- | --- | --- | --- | --- | --- |
| CRP, mg/L | 7.59 [4.00, 27.03] | 5.96 [3.14, 21.57] | 7.88 [3.14, 32.98] | <0.001 | 0.107 |
| PCT, ng/mL | 0.69 [0.40, 1.48] | 0.42 [0.19, 1.21] | 0.35 [0.14, 1.13] | <0.001 | 0.021 |
| IL-6, pg/mL | 3.47 [1.08, 52.95] | 7.99 [2.69, 25.19] | 11.64 [3.17, 41.84] | 0.208 | 0.123 |
| WBC, x10^9/L | 6.85 [5.50, 8.59] | 6.80 [5.41, 8.62] | 6.90 [5.49, 8.83] | 0.012 | 0.045 |
| ESR, mm/h | 50.00 [25.00, 97.00] | 48.00 [24.00, 83.00] | 44.00 [23.00, 78.00] | 0.017 | 0.098 |
| SAA, mg/L | 16.47 [7.70, 109.88] | 16.80 [6.00, 93.21] | 17.90 [6.70, 84.00] | 0.361 | 0.068 |

Data are presented as median (IQR). P-values were calculated using Kruskal-Wallis test. Abbreviations: PTX, parathyroidectomy; UC, unknown cause; CRP, C-reactive protein; IL-6, interleukin-6; WBC, white blood cell count; PCT, procalcitonin; ESR, erythrocyte sedimentation rate; SAA, serum amyloid A.

**Supplementary Table 3. Absolute event numbers during 3-year follow-up among CKD stages 4-5 patients with PTH < 150pg/mL.**

| Outcome | PTX(n=423) | Drug(n=10519) | UC(n=6233) |
| --- | --- | --- | --- |
| all-cause mortality, n | 52 | 2058 | 1834 |
| CVD mortality, n | 3 | 364 | 279 |
| Cardiovascular events, n | 32 | 1066 | 484 |
| Cerebrovascular events, n | 18 | 585 | 265 |

**Supplementary Table 4.** Landmark sensitivity analysis (≤300 days).

| Comparison | HR (95%CI) | P |
| --- | --- | --- |
| PTX(Reference) | 1 |  |
| Drug | 2.82 [0.95-8.35] | 0.061 |
| UC | 3.73 [1.26-11.05] | 0.017 |

PH assumption test: Group P = 0.188, Global P = 0.212

**Supplementary Table 5.** Covariate balance after inverse probability of weighting.

| covariable | PTX | Drug | UC | P value | unweighted SMD | weighted SMD |
| --- | --- | --- | --- | --- | --- | --- |
| age, year | 56.94 (12.47) | 58.47 (15.60) | 58.55 (15.77) | 0.711 | 0.550 | 0.076 |
| male | 277 (60.0%) | 6266 (59.5%) | 3685 (59.4%) | 0.957 | 0.227 | 0.009 |
| hypertension | 310 (67.3%) | 5261 (49.9%) | 3133 (50.1%) | 0.008 | 0.286 | 0.238 |
| diabetes | 190 (41.3%) | 2803 (26.6%) | 1706 (27.5%) | 0.071 | 0.352 | 0.209 |
| ACS | 64 (13.9%) | 1198 (11.4%) | 716 (11.5%) | 0.770 | 0.082 | 0.050 |
| Heart failure | 86 (18.6%) | 1841 (15.6%) | 960 (15.5%) | 0.722 | 0.123 | 0.055 |
| stroke | 85 (18.3%) | 1053 (10.0%) | 636 (10.2%) | 0.269 | 0.149 | 0.161 |
| dialysis | 180 (39.1%) | 4863 (46.2%) | 2845 (45.8%) | 0.332 | 1.070 | 0.095 |

Abbreviations: PTX, parathyroidectomy; UC, unknown causes; SMD, standardized mean difference; ACS, acute coronary syndrome.

**Supplementary Table 6.** Multivariate Cox regression for all-cause mortality in patients with persistently low PTH after 30 days following the index date.

| Parameters | Univariate analysis | | | |  | | Multivariate analysis | | | |
| --- | --- | --- | --- | --- | --- | --- | --- | --- | --- | --- |
|  | HR (95%CI) | | P | |  | | HR (95%CI) | | | P |
| age | 1.89(1.78-2.00) | | ＜0.001 | |  | 1.041(1.037-1.046) | | | | ＜0.001 |
| male | 1.29(1.17-1.43) | | ＜0.001 | |  | 1.230(1.106-1.367) | | | | ＜0.001 |
| SBP | 1.08(1.03-1.14) | | 0.001 | |  | 0.997(0.994-0.999) | | | | 0.039 |
| DBP | 0.88(0.84-0.93) | | ＜0.001 | |  | 1.006(1.001-1.010) | | | | 0.027 |
| dialysis | 0.91(0.83-1.01) | | 0.072 | |  |  | | | |  |
| hypertension | 1.29(1.17-1.43) | | ＜0.001 | |  | 0.998(0.880-1.131) | | | | 0.972 |
| diabetes | 1.78(1.60-1.97) | | ＜0.001 | |  | 1.245(1.098-1.412) | | | | 0.001 |
| ACS | 1.90(1.65-2.19) | | ＜0.001 | |  | 0.999(0.847-1.179) | | | | 0.993 |
| heartfailure | 1.91(1.70-2.15) | | ＜0.001 | |  | 1.379(1.193-1.593) | | | | ＜0.001 |
| arrhythmia | 1.72(1.41-2.09) | | ＜0.001 | |  | 0.986(0.796-1.222) | | | | 0.920 |
| hyperlipidemia | 1.13(0.95-1.34) | | 0.168 | |  |  | | | |  |
| stroke | 1.80(1.56-2.08) | | ＜0.001 | |  | 1.190(1.017-1.394) | | | | 0.030 |
| PVD | 1.76(1.23-2.51) | | 0.002 | |  | 0.852(0.582-1.247) | | | | 0.410 |
| malnutrition | 1.18(0.80-1.74) | | 0.403 | |  |  | | | |  |
| obesity | 0.83(0.59-1.18) | | 0.299 | |  |  | | | |  |
| fracture | 1.54(1.12-2.11) | | 0.007 | |  | 1.096(0.786-1.530) | | | | 0.588 |
| PTH | 0.96(0.92-1.01) | | 0.113 | |  |  | | | |  |
| P | 1.01(0.96-1.06) | | 0.650 | |  |  | | | |  |
| Ca | 0.84(0.80-0.89) | | ＜0.001 | |  | 0.855(0.698-1.048) | | | | 0.133 |
| ALP | 1.01(0.96-1.05) | | 0.760 | |  |  | | | |  |
| CK | 0.86(0.76-0.98) | | 0.021 | |  | | | 0.998(0.997-0.999) | 0.035 | |
| Cr | 0.84(0.79-0.88) | ＜0.001 | |  | | | | 1.001(0.999-1.002) | | 0.933 |
| BUN | 1.01(0.96-1.06) | | 0.684 |  | | | |  | |  |
| Cystatin C | 0.97(0.93-1.02) | | 0.196 |  | | | |  | |  |
| UA | 0.94(0.89-0.99) | | 0.018 |  | | | | 0.999(0.998-1.000) | | 0.194 |
| albumin | 0.75(0.71-0.79) | | ＜0.001 | |  | | | 0.950(0.939-0.962) | | ＜0.001 |
| total protein | 0.87(0.83-0.92) | | ＜0.001 | |  | | | 1.016(1.007-1.024) | | ＜0.001 |
| glucose | 1.08(1.05-1.12) | | ＜0.001 | |  | | | 1.009(0.997-1.020) | | 0.135 |
| LDL | 0.92(0.87-0.97) | | 0.002 | |  | | | 0.855(0.788-0.928) | | ＜0.001 |
| cholesterol | 0.94(0.90-0.99) | | 0.031 | |  | | | 1.119(1.050-1.923) | 0.001 | |
| triglyceride | 0.94(0.89-0.99) | | 0.027 | |  | | | 0.990(0.951-1.030) | 0.616 | |
| hemoglobin | 0.81(0.77-0.85) | ＜0.001 | | |  | | | 0.994(0.991-0.996) | ＜0.001 | |
| cause of low PTH |  | |  | |  | | |  | |  |
| PTX(Reference) | Reference | |  | |  | | | Reference | |  |
| Drug | 1.69(1.11-2.48) | | 0.009 | |  | | | 1.289(0.866-1.918) | | 0.212 |
| UC | 2.39(1.69-3.57) | ＜0.001 | | |  | | 1.567(1.041-2.359) | | | 0.031 |

Abbreviations: HR, hazard ratio; CI, confidence interval; SBP, systolic blood pressure; DBP, diastolic blood pressure; ACS, acute coronary syndrome; PVD, peripheral vascular disease; PTH, parathyroid hormone; P, phosphorus; Ca, calcium; ALP, alkaline phosphatase; CK, creatione phosphate kinase; NT-proBNP, N-terminal pro-B-type natriuretic peptide; Cr, creatinine; BUN, blood urea nitrogen; UA, uric acid; LDL, low-density lipoprotein cholesterol; PTX, parathyroidectomy; UC, unknown causes.

**Supplementary	Table 7.** Multivariate Cox regression for all-cause mortality in outpatients with persistently low PTH after 30 days post-index.

| Parameters | Univariate analysis | | | |  | | | | Multivariate analysis | |
| --- | --- | --- | --- | --- | --- | --- | --- | --- | --- | --- |
|  | HR (95%CI) | | P | |  | | | | HR (95%CI) | P |
| age | 1.92(1.79-2.05) | | ＜0.001 | |  | | | 1.041(1.035-1.046) | | ＜0.001 |
| male | 1.27(1.14-1.42) | | ＜0.001 | |  | | | 1.213(1.080-1.363) | | 0.001 |
| SBP | 1.13(1.07-1.19) | | ＜0.001 | |  | | | 0.999(0.997-1.003) | | 0.814 |
| DBP | 0.90(0.85-0.95) | | ＜0.001 | |  | | | 1.007(1.001-1.013) | | 0.018 |
| dialysis | 0.93(0.83-1.04) | | 0.193 | |  | | |  | |  |
| hypertension | 1.43(1.28-1.60) | | ＜0.001 | |  | | | 1.101(0.960-1.263) | | 0.168 |
| diabetes | 1.77(1.58-1.99) | | ＜0.001 | |  | | | 1.222(1.066-1.400) | | 0.004 |
| ACS | 1.83(1.57-2.13) | | ＜0.001 | |  | | | 0.938(0.787-1.117) | | 0.472 |
| heartfailure | 1.93(1.70-2.19) | | ＜0.001 | |  | | | 1.415(1.218-1.643) | | ＜0.001 |
| arrhythmia | 1.72(1.41-2.10) | | ＜0.001 | |  | | | 1.065(0.857-1.322) | | 0.571 |
| hyperlipidemia | 1.16(0.97-1.39) | | 0.095 | |  | | |  | |  |
| stroke | 1.78(1.53-2.07) | | ＜0.001 | |  | | | 1.130(0.957-1.334) | | 0.151 |
| PVD | 1.89(1.32-2.70) | | ＜0.001 | |  | | | 0.947(0.650-1.381) | | 0.778 |
| malnutrition | 1.17(0.73-1.87) | | 0.507 | |  | | |  | |  |
| obesity | 0.80(0.55-1.16) | | 0.237 | |  | | |  | |  |
| fracture | 1.32(0.93-1.87) | | 0.115 | |  | | |  | |  |
| PTH | 0.95(0.90-1.00) | | 0.060 | |  | | |  | |  |
| P | 0.99(0.94-1.04) | | 0.705 | |  | | |  | |  |
| Ca | 0.86(0.82-0.91) | | ＜0.001 | |  | | | 0.950(0.757-1.194) | | 0.661 |
| ALP | 0.99(0.94-1.04) | | 0.702 | |  | | |  | |  |
| CK | 0.84(0.72-0.97) | | 0.022 | |  | 0.999(0.997-1.000) | | | | 0.084 |
| Cr | 0.83(0.78-0.88) | ＜0.001 | |  | | | 0.999(0.998-1.000) | | | 0.784 |
| BUN | 0.98(0.93-1.04) | | 0.540 |  | | |  | | |  |
| Cystatin C | 0.98(0.93-1.03) | | 0.451 |  | | |  | | |  |
| UA | 0.91(0.86-0.97) | | 0.002 |  | | | 0.998(0.997-0.999) | | | 0.007 |
| albumin | 0.76(0.72-0.80) | | ＜0.001 | |  | 0.953(0.940-0.966) | | | | ＜0.001 |
| total protein | 0.87(0.82-0.92) | | ＜0.001 | |  | 1.016(1.006-1.025) | | | | 0.001 |
| glucose | 1.08(1.04-1.12) | | ＜0.001 | |  | 0.993(0.990-1.020) | | | | 0.221 |
| LDL | 0.96(0.90-1.02) | | 0.172 | |  | | |  | |  |
| cholesterol | 1.00(0.94-1.06) | | 0.957 | |  | | |  | |  |
| triglyceride | 0.97(0.92-1.03) | | 0.314 | |  | | |  | |  |
| hemoglobin | 0.80(0.76-0.85) | ＜0.001 | |  | | |  | | |  |
| cause of low PTH |  | |  |  | | |  | | |  |
| PTX(Reference) | Reference | |  |  | | | Reference | | |  |
| Drug | 1.72(1.32-2.59) | | 0.011 |  | | | 1.349(0.882-2.064) | | | 0.168 |
| UC | 2.41(1.58-3.68) | ＜0.001 | |  | | | 1.595(1.032-2.464) | | | 0.036 |

Abbreviations: HR, hazard ratio; CI, confidence interval; SBP, systolic blood pressure; DBP, diastolic blood pressure; ACS, acute coronary syndrome; PVD, peripheral vascular disease; PTH, parathyroid hormone; P, phosphorus; Ca, calcium; ALP, alkaline phosphatase; CK, creatione phosphate kinase; NT-proBNP, N-terminal pro-B-type natriuretic peptide; Cr, creatinine; BUN, blood urea nitrogen; UA, uric acid; LDL, low-density lipoprotein cholesterol; PTX, parathyroidectomy; UC, unknown causes.

**Supplementary Figures**

**Supplementary Figure 1.** Kaplan-Meier curves for cardiovascular and cerebrovascular events in overall (a, b), dialysis (c, d), and non-dialysis (e, f) subgroups. Abbreviations: PTX, parathyroidectomy; UC, unknown causes.

**Supplementary Figure 2.** Cross-validation curves for selecting the optimal λ.

**Supplementary Figure 3.** Kaplan-Meier curves comparing different drug types with PTX and UC groups. (a) Calcimimetics, (b) Vitamin D and its analogs, (c) Phosphate binders. Abbreviations: PTX, parathyroidectomy; UC, unknown causes; VD, Vitamin D and its analogs; P binders, phosphate binders.

**Supplementary Figure 4.** Kaplan-Meier curves for 30-day post-baseline PTH status. (a) PTX group, (b) Drug group, (c) UC group: comparison of three PTH status categories — persistently low (low), no longer low (non-low), and missing (NA). (d) Comparison of survival in persistently low PTH patients across the three groups. Abbreviations: PTX, parathyroidectomy; UC, unknown causes; NA, not available.

**Supplementary Figure 5.** Kaplan-Meier curves for all-cause (A) and cardiovascular (B) mortality restricted to outpatients with persistently low PTH after 30 days post-index. Abbreviations: PTX, parathyroidectomy; UC, unknown causes.
